# High Physical Activity Level May Reduce Menopausal Symptoms

**DOI:** 10.3390/medicina55080466

**Published:** 2019-08-11

**Authors:** Magdalena Dąbrowska-Galas, Jolanta Dąbrowska, Kuba Ptaszkowski, Ryszard Plinta

**Affiliations:** 1 Department of Kinesitherapy and Special Methods, School of Health Sciences in Katowice, Medical University of Silesia, 40-752 Katowice, Poland; 2 Department of Kinesiology, School of Health Sciences in Katowice, Medical University of Silesia, 40-752 Katowice, Poland; 3 Department of Clinical Biomechanics and Physiotherapy in Motor System Disorders, Faculty of Health Science, Wroclaw Medical University, 50-355 Wroclaw, Poland; 4 Department of Adapted Physical Activity and Sport, School of Health Sciences in Katowice, Medical University of Silesia, 40-752 Katowice, Poland

**Keywords:** IPAQ, MRS, middle-aged, physical activity

## Abstract

*Background and Objectives:* Menopause is a normal physiological change occurring at a woman’s mid-life. During this time, women experience vasomotor, physical and physiological problems, which reduce their quality of life. Many women are searching for different, alternative methods to reduce the severity of menopausal symptoms. Physical activity (PA) is one of the recommended methods to reduce menopausal symptoms. The purpose of this study was to investigate the association between specific domains (physical activity during leisure time, at work, during transportation and household activities) and the menopausal symptoms. *Materials and Methods:* We included 305 women aged 40–65 in the study. All participants were divided into three groups according to menopausal status. The research tools used were the International Physical Activity Questionnaire (IPAQ) to assess physical activity level in four domains and the Menopause Rating Scale (MRS) to assess the severity of menopausal symptoms. The data analysis was performed by Chi-square test and analysis of variance (ANOVA) with post hoc Tuckey test. *Results:* Menopausal stage was significantly associated with the total MRS score (*p* < 0.001) and specifically the urogenital and somato–vegetative subscores (*p* < 0.001). Physical activity was significantly associated (*p* < 0.001) with leisure time (according to IPAQ domains). Most postmenopausal women had high PA level (59.66%). Significantly less women with high PA levels had severe urogenital symptoms: 10.82% of participant with a low PA level, 11.15% with a moderate PA level and 4.26% with a high PA level (*p* = 0.046). *Conclusions:* Physical activity during leisure time is associated to menopausal symptoms in Polish women. Women with high and moderate PA levels have less severe menopausal symptoms compared to inactive women. Middle-aged women with low PA levels at work suffer from more severe somato–vegetative symptoms.

## 1. Introduction

Menopause is a normal physiological change occurring at a woman’s mid-life. During this time women experience vasomotor, physical and physiological problems reducing their quality of life [1]. The mean age of menopause in Europe is 51.3 [2]; in America, 52.5 years [3]; while in Latin America it is 48.3 [4]. The age of the last period is affected by poor physical activity, lower level of education, hormonal contraception, smoking and alcohol intake [3].

It has been shown that the use of hormone therapy (HT) alleviates menopausal symptoms [5]. However many women are searching for different, alternative methods to reduce the severity of menopausal symptoms [5,6]. Nonpharmaceutical intervention such as physical activity was proven to be an effective method of reducing menopausal symptoms, decreasing bone loss and increasing muscle strength in menopausal women [5,6,7,8].

PA is defined as a behavior that involves human movement, resulting in physiological attributes including increased energy expenditure and improved physical fitness [5]. The benefits of PA are well-established, however, most midlife women are not sufficiently physically active to meet Physical Activity Guidelines [9]. According to the World Health Organization (WHO) Guidelines, adults should engage in at least 150 min of moderate intensity (activity types between 3–6 metabolic equivalent of task METs - defined as the amount of oxygen consumed while sitting at rest and is equal to 3.5 ml O2 per kg body weight x min) per week, or at least 75 min of vigorous intensity (activity types 6 METs) per week, or an equivalent combination of moderate to vigorous intensity PA. Additionally, all adults should perform moderate or high-intensity muscle strengthening activities that involve all major muscle groups on two or more days of the week [10]. Previous studies have shown a reduction in daily energy expenditure in women and a shift toward a more sedentary lifestyle during the menopausal transition [11,12].

Regular physical activity, including resistance exercises, aerobic training, stretching exercises and relaxation promotes well-being, prevents osteoporosis by keeping cartilage tissue and bones stronger, decreases the risk of chronic diseases and helps to maintain proper body weight and prevent gradual body weight gain [13,14,15].

In 2018 The Scientific Advisory Committee of the Royal College of Obstetricians and Gynaecologists published information that lifestyle changes such as regular aerobic exercise, e.g., running and swimming or low-intensity exercise, such as yoga, as well as reducing intake of caffeine and alcohol may also help to reduce hot flushes and night sweats [16]. Resistance-training programs were proven to decrease the frequency of moderate and severe hot flushes among postmenopausal women and could be an effective and safe treatment option to alleviate vasomotor symptoms [17]. Other study suggested that higher doses of impact exercise attenuated natural age-related declines in total bone mineral density in postmenopausal women [18]. Physical activity, especially aerobic dance, was proven as an effective and efficient method in improving health, physical function and physical fitness in postmenopausal women [8]. The systematic review and meta-analysis published in 2018 recommended yoga for menopausal women. Based on the findings, yoga seems to improve all menopausal symptoms when compared to no intervention, and it seems to be at least as effective as other forms of exercise. Since many menopausal women request complementary therapies in addition to or instead of HT, yoga can be clearly recommended as an adjunct intervention for menopausal women [19]. However, a study with six months moderate physical activity intervention showed that exercise was not an effective treatment for hot flashes and night sweats but a definitive statement could not be made until more evidence was available [20].

Although evidence suggests the benefits of PA on menopausal symptoms, there have been some inconsistencies regarding the type or intensity of exercise and the severity of menopausal symptoms [13,21,22]. Physical activity is usually identified with organized exercises, trainings or any form of activity during free time. Physical activity is performed during different day activities. Little light has been shed on the effect of physical activity during household activities or at work. Therefore, the purpose of this study was to investigate the association between specific domains (physical activity during leisure time, at work, during transportation and household activities) and menopausal symptoms.

## 2. Materials and Methods

The study protocol was reviewed and approved by the Bioethical Committee of the Medical University of Silesia in Katowice (KNW/002/KB1/112/14, date of approval: 30 September 2014). All women gave their informed consent to participate in the study.

### 2.1. Study Participants

This was a cross-sectional study, carried out in 2014. Subjects were selected among the middle-aged women (aged 40–65) who visited women’s health clinics in Silesia, Poland, and agreed to participate in this study. The main investigator met with the physicians in each of the selected clinics to discuss the aim of the study and the data collection protocol. Participants who met the criteria were informed about the study. Guidance on how to answer questions in each section was given to all participants. Delivery and return of the questionnaire took place in the clinic.

The inclusion criteria were age of 40–65; no serious illness and the consent to participate in the research. Exclusion criteria were physical problems related to spinal cord injury, paralysis, history of antidepressant use, history of psychiatric disorders, history of hormonal therapy (HT) use, no symptoms for all items of the MRS. All participants were divided into three groups according to menopausal status on the basis of Stages of Reproductive Aging Workshop (STRAW) criteria, as follows: premenopause is defined as having regular menstrual periods; perimenopause is characterized by persistent ≥ 7 days difference in length of consecutive cycles or interval of amenorrhea of ≥ 60 days; and postmenopause is the period after 12 consecutive months of amenorrhoea [23]. We excluded 21 women who had history of HT use and 61 subjects with missing data from the analysis. Therefore, 305 women were finally eligible for the study. The average age of the study group was 48.47 ± 6.32.

### 2.2. Methods

The research tool was a questionnaire that was voluntarily and anonymously completed by the respondents in the research group.

The questionnaire included questions about age, educational level, marital status, body mass, height, general health, relations with a partner, educational level, tobacco and alcohol use. The second part consisted of the Menopause Rating Scale (MRS) to assess menopausal symptoms. The scale ensures documented credibility, sensitivity, reliability and duplication of results. The scale has been standardized and translated into more than 27 languages including Polish, to differentiate among the menopausal symptoms in women. The MRS was developed in the early 1990s to measure the severity of menopause-related complaints by rating a profile of symptoms and is used in most countries [24,25,26,27,28]. Subjective complaints in each of the 11 items are classified into three domains: psychological (4 symptoms: depressed, irritable, anxious, exhausted), somato–vegetative (four symptoms: sweating or hot flushes, cardiac complaints, sleeping disorders, joint and muscle complaints), urogenital symptoms (three symptoms: sexual problems, urinary problems and vaginal dryness). Severity of each of the symptoms ranges from 0 (absent) to 4 (very severe). Total score is the sum in each of the domains (psychological 0–16 points, somato–vegetative 0–16 point, urogenital 0–12 points) and in total 0 (asymptomatic) to 44 (highest degree of complaints) [24].

The third part of the questionnaire consisted of the long form of the International Physical Activity Questionnaire (IPAQ) to assess the physical activity level in four domains: transportation (to travel from place to place), work, domestic chores and leisure-time including vigorous and moderate activity and walking during previous week. IPAQ was utilized to estimate PA levels. This self-reported measurement has shown reliability and validity within different contexts (Craig et al., 2003), including with the Polish adolescent population [29,30,31].

Walking meant marching, Nordic walking, walking. Moderate physical activity meant average effort with a slightly increased breathing rate and heart beat (e.g., swimming, yoga or recreational cycling). Vigorous physical activity meant hard effort, causing an increased breathing rate and heart beat (e.g., running, aerobics, sport cycling, tennis). In accordance with the IPAQ methodology, only activities lasting individually longer than 10 min were taken into consideration [29].

Continuous Score IPAQ results were expressed as MET-min per week and calculated by multiplying the MET assigned to it (vigorous—8 MET, moderate—4MET and walking—3.3 MET) by the number of days it was performed during a week, where MET corresponds to O_2_ consumption during the rest and equals 3.5 mL O_2_/kg of the body mass per minute.

Physical activity level in all domains and in total was divided into low, moderate and high according to IPAQ methodology:

High PA level:

- Vigorous-intensity activity on at least three days and accumulating at least 1500 MET-minutes/week, or
- Seven or more days of any combination of walking, moderate-intensity or vigorous intensity activities achieving a minimum of at least 3000 MET-minutes/week

Moderate PA level:

Any one of the following three criteria:

- Three or more days of vigorous activity of at least 20 min per day OR
- Five or more days of moderate-intensity activity or walking of at least 30 min per day or
- Five or more days of any combination of walking, moderate-intensity or vigorous intensity activities achieving a minimum of at least 600 MET-min/week.

Low PA level:

This is the lowest level of physical activity. Those individuals who not meet criteria for categories 2 or 3 are considered low/inactive [32].

### 2.3. Statistical Analysis

The statistical calculations were conducted using STATISTICA software (Statistica v10, StatSoft, Krakow, Poland). Data was expressed as means, standard deviations (SD) and percentages. Shapiro-Wilk test was used to determine normality of data distribution. The data analysis was performed by Chi-square test and analysis of variance (ANOVA) with post hoc Tuckey test. The value of *p* < 0.05 was assumed as statistically significant.

## 3. Results

Characteristics of the sample across the three menopausal stages are presented in Table 1. As expected, the postmenopausal women were significantly older than the perimenopausal women (Tukey test *p* < 0.001), and the premenopausal women were the youngest (Tukey test *p* < 0.001). There were no significant differences between the groups in marital status and tobacco smoking, however, most women were married (85.05% in premenopause, 82.28% in perimenopause and 84.87% in postmenopause), nonsmokers (75.70%, 81.01%, and 86.55% respectively). No significant differences between the groups were observed in place of living and alcohol consumption. Postmenopausal women were significantly better educated, 39.50% of them had high educational level (*p* < 0.001). No statistically significant differences were observed in material status between the groups. “Very good” material status was, however, reported mostly by postmenopausal women (*p* = 0.082).

Table 2 shows the severity of menopausal symptoms for each MRS domain in the three menopausal stages. Menopausal stage was significantly associated with the total MRS score (*p* < 0.001) and specifically the urogenital and somato–vegetative subscores (*p* < 0.001). Most perimenopausal women had severe urogenital symptoms (37.97%), somato–vegetative symptoms (32.91%) and menopausal symptoms in total (43.04%) according to MRS comparing to pre- and postmenopausal women. The menopausal stage was not significantly associated with the psychological symptoms.

Table 3 shows the physical activity level for each IPAQ domain in the three menopausal stages.

Physical activity was significantly associated (*p* < 0.001) with the leisure time (according to IPAQ domains). Most postmenopausal women had high PA level (59.66%). Premenopausal women were mostly inactive (72.90%). Most women in all menopausal stages were inactive in remaining IPAQ domains, however, physical activity level was not significantly associated with those domains (household and gardening, transportation, work).

The physical activity level of each IPAQ domain according to the severity of menopausal symptoms in MRS domains is presented in Table 4. A significant difference was observed between leisure time domain and total menopausal symptoms (*p* = 0.019) and urogenital symptoms (*p* = 0.046). 2.98% of women with high PA levels had severe menopausal symptoms compared to groups with low PA levels (10.2%) and moderate PA levels (9.87%). Significantly less women with high PA levels had severe urogenital symptoms: 10.82% of participant with low PA levels, 11.15% with moderate PA levels and 4.26% with high PA levels (*p* = 0.046). Physically active women at work had significantly less severe somato–vegetative symptoms (*p* = 0.002). Less participants of the moderately active group compared to the inactive group had severe somato–vegetative symptoms (6.89% vs. 8.85%), moderate symptoms (6.56% vs. 18.69%) and mild symptoms (7.21% vs. 21.31%). No significant differences were observed between other domain. 

## 4. Discussion

This study examined the association between physical activity in four domains and menopausal symptoms among women aged 40 to 65 in Poland.

Middle-aged women experience a wide spectrum of menopause-related symptoms with vasomotor and urogenital symptoms being the most common [3]. Based on the present study, according to MRS, the most frequently reported symptoms were urogenital and somato–vegetative symptoms, especially among perimenopausal women. These data are similar to the results of other studies using the same tools [33] The highest percentage of somato–vegetative symptoms were also presented by other authors [34,35] but unlike to the present study, the severity of menopausal symptoms among menopausal stages was not compared.

The results of the study from Asia showed that more severe physical and physiological symptoms were observed in perimenopausal women while urogenital symptoms were observed in postmenopausal women [36,37,38,39]. In Brazil, muscle and joint discomfort was most common in postmenopausal women, but the authors suggested that it was combined with aging process and osteomuscular diseases [40].

Comparing the severity of menopausal women, as assessed by MRS, women from Latin America had more severe somato–vegetative, psychological and urogenital symptoms compared to Europe and Asia. Europeans and Asians reported fewer menopausal symptoms, while Asians had much milder or even lack of symptoms [4].

The reason for the differences in the severity of menopausal symptoms can be justified by racial differences, climate, genetics, geographic area and age for menopause.

A study conducted on Polish women aged 45–55 showed that most women were moderately active in all IPA domains (work, transportation, leisure time and household) [41]. In that study, participants were not divided according to menopausal stage.

Women tend to present a more rapid decline in physical fitness than men in middle age, and the level of physical activity decreases with age [15]. A cross-sectional study conducted on 2204 perimenopausal women, where short version of IPAQ was used, showed that 58% of women had low physical activity and 28% moderate PA levels [42]. In our study IPAQ results were divided into four domains (work, transportation, leisure time and household). In leisure time peri- and postmenopausal women had mostly high physical activity levels, while premenopausal women had low PA levels. In the household IPAQ domain, all menopausal women had low to moderate PA levels. At work and during transportation, women had low PA levels.

In our study only 10% of women had higher education, while in postmenopausal group almost 40% had university education. In this group the level of PA in leisure time was the highest. Higher education is usually associated with greater knowledge about health and higher income. These women have better access to health care services, fitness centers and medical advice, which can lead to a higher PA level and a better level of their health [43].

Some authors pointed out that one of the independent factors associated with severity of menopausal symptoms was physical activity [35].

Significant differences were observed between the PA level in the leisure time domain and the total MRS score, the urogenital subscore and PA level at work, and somato–vegetative subscore. The lowest percentage of women highly active had severe urogenital symptoms and menopausal symptoms in total. Furthermore, women moderately active had less severe and moderate symptoms than inactive women.

Our results are in line with previous studies showing that moderate and high PA levels in all IPAQ domains are associated with less severe menopausal symptoms, while the most inactive women had severe menopausal symptoms [13]. In our results, no statistically significant differences were observed in menopausal symptoms in total, and such IPAQ domains as transportation, work and household. However, inactive women had the most severe menopausal symptoms.

Our results are consistent with several previous studies, which showed that the habitual physical activity of at least 60 min/day had a positive influence on the menopausal symptoms [21].

We did not observe an association between leisure time, physical activity and vasomotor and psychological domain. A cohort study of 631 women showed that moderate activity was significantly associated with improved psychosocial and physical symptoms but did not associate with changes in vasomotor and sexual symptoms. Similarly, many large studies on this topic also did not show the significant association between PA and vasomotor symptoms [44,45,46].

Several previous studies showed that physical activity correlates with psychological symptoms [42,45,46,47]. In our studies no significant differences between these variables were observed.

A previous study indicated that the relationship between physical activity and menopausal symptoms had a U-shaped trend, and moderate level of physical activity was significantly associated with reduced menopausal symptoms. Highly active women experienced more symptoms than the moderately active groups [42].

Earlier studies also reported that regular physical activity might significantly reduce menopausal symptoms, improve general well-being and health status [14,48,49,50]. In another study, a moderate physical activity level was associated with less severe menopausal symptoms, a lower level of physical disorders, better general health and social functioning [51].

Although many studies have reported that physical activity improves menopausal symptoms, the relationship between these variables, according to PA dose and validated self-reported questionnaires, has not been well studied.

To our knowledge, this is one of few studies which investigated the association between physical activity and menopausal symptoms using validated instruments in Polish middle-aged women.

This study stands out for being the first carried out in Europe employing IPAQ as the instrument for assessing physical activity level not only in leisure time but also at work, in the household and during transportation, according to menopausal stages and menopausal symptoms assessed by MRS.

The limitations of the study should, however, be recognized. First, all participants were recruited from women’s health clinics, which means that women who did not visit a doctor regularly were not included and our results may not be generalizable to all middle-age women in Poland. Self-reported questionnaires were used, however, these are the usual methods in the literature. Further, longitudinal research is warranted to explain other factors influencing severity of menopausal symptoms and to achieve higher level of evidence.

## 5. Conclusions

The present study showed that perimenopausal women experience more severe symptoms than pre and postmenopausal women. The level of peri and postmenopausal women is satisfying, while premenopausal women are inactive. Physical activity during leisure time is associated with menopausal symptoms in Polish women. Women with high and moderate PA levels have less severe menopausal symptoms compared to inactive women. Middle-aged women with low PA levels at work suffer from more severe somato–vegetative symptoms. Motivating women to be more active at work can reduce somato–vegetative symptoms, while motivating them to an increase physical activity in leisurely times can reduce the severity of menopausal symptoms. A further randomized study will be carried out to assess the appropriate dose of PA for middle-aged women, however the results suggest that lack of physical activity leads to increased severity of menopausal symptoms.

## Figures and Tables

**Table 1:** Characteristic of the sample according to menopause status.

	Premenopausal Women %	N = 107	Perimenopausal Women %	N = 79	Postmenopausal Women %	N = 119	*p*
Age (mean ± sd)	42.89	1.62	45.97	2.55	55.13	4.38	*p* < 0.0001 *
BMI (mean ± sd)	24.86	3.64	25.44	3.72	25.84	3.193	*p* = 0.112
Marital status							
Married	85.05%	91	82.28%	65	84.87%	101	*p* = 0.283
Single	5.61%	6	7.59%	6	2.52%	3	
Cohabitating	0.93%	1	0.00%	0	0.84%	1	
Divorced	6.54%	7	8.86%	7	5.04%	6	
Widow	1.87%	2	1.27%	1	6.72%	8	
Place of living							
Village	10.28%	11	11.39%	9	12.61%	15	*p* = 0.908
City up to 100 thous. inhabitants	34.58%	37	37.97%	30	31.93%	38	
City over 100 thous. inhabitants	55.14%	59	50.63%	40	55.46%	66	
Education							
Primary	38.32%	41	39.24%	31	30.25%	36	*p* < 0.0001 *
Secondary	51.40%	55	32.91%	26	30.25%	36	
Higher	10.28%	11	27.85%	22	39.50%	47	
Material status	0.00%		0.00%		0.00%		
Average	4.67%	5	1.27%	1	3.36%	4	*p* = 0.082
Good	54.21%	58	51.90%	41	38.66%	46	
Very good	41.12%	44	46.84%	37	57.98%	69	
Alcohol consumption							
No	24.30%	26	27.85%	22	27.73%	33	*p* = 0.806
Yes	75.70%	81	72.15%	57	72.27%	86	
Tabacco use							
No	75.70%	81	81.01%	64	86.55%	103	*p* = 0.112
Yes	24.30%	26	18.99%	15	13.45%	16	
Chi2 test or analysis of variance							

* *p* < 0.05.

**Table 2:** The evaluation of the severity of menopausal symptoms (Menopause Rating Scale (MRS) scale) according to menopause status.

	Premenopausal Women	107	Perimenopausal Women	79	Postmenopausal Women	119	*p*
MRS-TOTAL							
None	36.79%	39	6.33%	5	6.72%	8	*p* < 0.0001 *
Mild	25.47%	27	16.46%	13	36.13%	43	
Moderate	23.58%	25	34.18%	27	39.50%	47	
Severe	14.15%	15	43.04%	34	17.65%	21	
MRS-UROGENITAL							
None	39.25%	42	8.86%	7	15.13%	18	*p* < 0.0001 *
Mild	22.43%	24	11.39%	9	26.05%	31	
Moderate	20.56%	22	41.77%	33	32.77%	39	
Severe	17.76%	19	37.97%	30	26.05%	31	
MRS-SOMATO-VEGETATIVE							
None	55.14%	59	7.59%	6	23.53%	28	*p* < 0.0001 *
Mild	20.56%	22	29.11%	23	35.29%	42	
Moderate	18.69%	20	30.38%	24	27.73%	33	
Severe	5.61%	6	32.91%	26	13.45%	16	
MRS-PSYCHOLOGICAL							
None	24.30%	26	18.99%	15	22.69%	27	*p* = 0.345
Mild	30.84%	33	32.91%	26	26.05%	31	
Moderate	28.97%	31	20.25%	16	24.37%	29	
Severe	15.89%	17	27.85%	22	26.89%	32	
Chi2 test							
MRS-Menopause Rating Scale							

* *p* < 0.05.

**Table 3:** Physical activity level in domains (International Physical Activity Questionnaire (IPAQ) scale) according to menopause status.

	Premenopausal Women	107	Perimenopausal Women	79	Postmenopausal Women	119	*p*
IPAQ-Leisure							*p* < 0.0001 *
Low-level PA	72.89%	78	12.66%	10	21.85%	26	
Moderate-level PA	6.54%	7	40.51%	32	18.49%	22	
High-level PA	20.5 %	22	46.84%	37	59.66%	71	
IPAQ-Houshold and gardening							*p* = 0.295
Low-level PA	40.19%	43	34.18%	27	42.86%	51	
Moderate-level PA	38.32%	41	32.91%	26	36.97%	44	
High-level PA	21.50%	23	32.91%	26	20.17%	24	
IPAQ-Transportation							*p* = 0.545
Low-level PA	69.16%	74	67.09%	53	73.95%	88	
Moderate-level PA	30.84%	33	32.91%	26	26.05%	31	
IPAQ-Work							*p* = 0.584
Low-level PA	60.75%	65	67.09%	53	66.39%	79	
Moderate-level PA	39.25%	42	32.91%	42	33.61%	40	
Chi2 test							
IPAQ-International Physical Activity Questionnaire							

* *p* < 0.05.

**Table 4:** Physical activity level in domains (IPAQ scale) according to severity of menopausal symptoms (MRS scale).

	IPAQ									
	Work		Transport			Household			Leisure	
	Low-level PA	Moderate-level PA	Low-level PA	Moderate-level PA	Low-level PA	Moderate-level PA	High-level PA	Low-level PA	Moderate-level PA	High-level PA
MRS - TOTAL										
None	9.87%	7.24%	11.84%	5.26%	6.25%	6.25%	4.61%	10.53%	4.28%	2.30%
Mild	15.79%	11.51%	21.71%	5.59%	11.51%	9.87%	5.92%	12.50%	11.51%	3.29%
Moderate	23.36%	9.21%	20.72%	11.84%	14.47%	11.51%	6.58%	5.59%	9.87%	17.11%
Severe	15.46%	7.57%	16.45%	6.58%	7.57%	8.88%	6.58%	10.20%	9.87%	2.96%
*p*	*p* = 0.163		*p* = 0.134		*p* = 0.765			*p* = 0.019 *		
MRS-UROGENITAL										
None	12.13%	9.84%	14.75%	7.21%	7.87%	8.85%	5.25%	12.13%	5.90%	3.93%
Mild	12.79%	8.20%	16.07%	4.92%	6.89%	7.54%	6.56%	9.51%	8.85%	2.62%
Moderate	22.30%	8.52%	21.31%	9.51%	13.77%	10.49%	6.56%	10.82%	16.72%	3.28%
Severe	17.38%	8.85%	18.36%	7.87%	11.15%	9.51%	5.57%	10.82%	11.15%	4.26%
*p*	*p* = 0.138		*p* = 0.664		*p* = 0.657			*p* = 0.046 *		
MRS-SOMATO-VEGETATIVE										
None	15.74%	14.75%	23.93%	6.56%	11.48%	10.82%	8.20%	16.07%	10.16%	4.26%
Mild	21.31%	7.21%	19.34%	9.18%	11.15%	11.80%	5.57%	10.49%	14.10%	3.93%
Moderate	18.69%	6.56%	17.05%	8.20%	10.49%	7.87%	6.89%	10.49%	11.48%	3.28%
Severe	8.85%	6.89%	10.16%	5.57%	6.56%	5.90%	3.28%	6.23%	6.89%	2.62%
*p*	*p* = 0.002 *		*p* =0.232		*p* = 0.806			*p* = 0.397		
MRS-PSYCHOLOGICAL										
None	14.43%	7.87%	15.08%	7.21%	9.51%	7.21%	5.57%	9.51%	9.51%	3.28%
Mild	19.02%	10.49%	21.64%	7.87%	14.75%	8.20%	6.56%	14.43%	10.49%	4.59%
Moderate	15.41%	9.51%	17.38%	7.54%	8.85%	10.49%	5.57%	10.16%	11.48%	3.28%
Severe	15.74%	7.54%	16.39%	6.89%	6.56%	10.49%	6.23%	9.18%	11.15%	2.95%
*p*	*p* = 0.911		*p* = 0.888		*p* = 0.124			*p* = 0.804		
MRS-Menopause Rating Scale										
IPAQ-International Physical Activity Questionnaire										
Chi2 test										

* *p* < 0.05.
